# Референсные интервалы тиреотропного гормона у беременных женщин, проживающих в центральных регионах РФ

**DOI:** 10.14341/probl13151

**Published:** 2022-12-20

**Authors:** А. А. Рыбакова, Н. М. Платонова, Н. М. Малышева, Л. В. Никанкина, А. Р. Елфимова, Е. А. Трошина

**Affiliations:** Национальный медицинский исследовательский центр эндокринологии; Национальный медицинский исследовательский центр эндокринологии; Национальный медицинский исследовательский центр эндокринологии; Национальный медицинский исследовательский центр эндокринологии; Национальный медицинский исследовательский центр эндокринологии; Национальный медицинский исследовательский центр эндокринологии

**Keywords:** тиреотропный гормон, беременность, йодурия, йодный дефицит

## Abstract

ОБОСНОВАНИЕ. Беременность — состояние, при котором происходят важные структурные и физиологические изменения щитовидной железы. В связи с этим экспертами Американской и Европейской тиреоидологических ассоциаций рекомендована разработка специфических референсных интервалов тиреоидных гормонов у беременных женщин с учетом природных и социально географических особенностей исследуемого региона.ЦЕЛЬ. Предложить референсные интервалы для тиреотропного гормона (ТТГ) у беременных женщин, проживающих в условиях легкого дефицита йода (в центральных регионах РФ).МАТЕРИАЛЫ И МЕТОДЫ. В наблюдательное многоцентровое поперечное сплошное исследование были включены 2008 здоровых беременных женщин на разных сроках беременности, проживающих в трех сопоставимых по степени тяжести дефицита йода регионах РФ (Москва, Иваново, Смоленск). У всех беременных женщин определяли уровни ТТГ, антител к тиреоидной пероксидазе, антител к тиреоглобулину в сыворотке крови, экскреции йода с мочой в утренней порции мочи (церий-арсенитным методом). Женщины, имеющие повышенный титр антител к тиреопероксидазе и/или антител к тиреоглобулину по результатам обследования, исключались из исследования (245 женщин). Исследование проводилось с августа 2018 по декабрь 2020 гг., конечным результатом исследования явилась оценка верхнего и нижнего уровней ТТГ и ее сопоставление с йодной обеспеченностью беременных женщин. Результаты оценивались с помощью расчета 2,5 и 97,5 процентилей. Статистически значимыми предполагались различия при р<0,05.РЕЗУЛЬТАТЫ. Нами подтверждено наличие йодного дефицита на исследуемых территориях. Медианная концентрация йода в моче составила в Москве 106 мкг/л, в Иваново — 119 мкг/л, в Смоленске — 134 мкг/л. Пациенткам при первичной постановке на учет в женской консультации был рекомендован прием физиологических доз калия йодида 150–200 мкг в сутки. Женщины были разделены на 2 группы в соответствии с уровнем йодурии. В группе с оптимальным уровнем йодурии уровень ТТГ составил в I триместре 0,006–3,36, во II триместре — 0,20–3,74, в III триместре — 0,33–3,68 мМЕ/л. В группе с легким йодным дефицитом: в I триместре — 0,11–3,00, во II триместре — 0,22–3,78, в III триместре — 0,07–3,04 мМЕ/л. При статистическом анализе данных было выявлено, что при сопоставлении уровней ТТГ по триместрам в зависимости от места проживания статистической разницы не выявлено (p=0,239).ЗАКЛЮЧЕНИЕ. По результатам нашего исследования получены данные о том, что уровень ТТГ у здоровых беременных женщин, проживающих в центральных регионах РФ, сопоставимых по степени тяжести природного йодного дефицита, не превышает 3,8 мМЕ/л во всех триместрах.

## Abstract (en)

BACKGROUND: Pregnancy is a condition with important structural and physiological changes in the thyroid gland. In this regard, experts of thyroid associations have recommended developing specific reference intervals taking into account the natural and socio-geographical characteristics of the region under study.AIM: To conduct an epidemiological analysis and evaluate TSH reference intervals in pregnant women living in the central regions of the Russian Federation with mild iodine deficiency.MATERIALS AND METHODS: We have conducted the observational multicenter cross-sectional study included 2008 healthy pregnant women at different trimesters of pregnancy, from three regions of the Russian Federation (Moscow, Ivanovo and Smolensk). We assessed the level of thyroid-stimulating hormone, antibodies to thyroid peroxidase, antibodies to serum thyroglobulin, the level of iodine concentration in the morning portion of urine (cerium arsenic method) and we have conducted a questionnaire (date birth and gestational age). Women with elevated titers of anti-TPO and/or anti-TG antibodies were excluded from the study (245 women). As a result, we assessed high and medium levels of TSH and its overestimation with iodine sufficiency in pregnant women. The results are presented using the calculation of 2.5 and 97.5 percentiles.RESULTS: We confirmed the presence of iodine deficiency in the study areas. The median concentration of iodine in the urine was: in Moscow 106 μg/l, in Ivanovo 119 μg/l, in Smolensk 134 μg/l. Pregnant women were divided into 2 groups according to iodine adequacy. In the group with optimal iodine supply, the level of TSH was 0,006–3,36 in the 1st trimester, 0,20–3,74 in the 2nd trimester, and 0,33–3,68 mIU/L in the 3rd trimester. In the group with mild iodine deficiency — in the 1st trimester it was 0,11–3,00, in the 2nd trimester 0,22–3,78, in the 3rd trimester 0,07–3,04 mIU/l. Statistical analysis of the data revealed that when comparing the level of TSH by trimester, depending on the place of residence, no statistical difference was found (p = 0,239).CONCLUSION: We obtained that the level of TSH in healthy pregnant women living in the central regions of the Russian Federation does not exceed 3.8 mIU/l in all trimesters.

## ВВЕДЕНИЕ

Во время беременности происходят физиологические и морфологические изменения в структуре и функции щитовидной железы. В первую очередь это связано с возрастающей потребностью в тиреоидных гормонах. Происходят физиологическое повышение уровня тироксинсвязывающего глобулина, стимуляция щитовидной железы высокими концентрациями хорионического гонадотропина, увеличение дейодирования йодтиронинов матери за счет функционирования дейодиназы 3-го типа для обеспечения повышенной потребности в тиреоидных гормонах. Все это ведет к повышению уровня общих фракций тироксина и трийодтиронина и закономерному снижению уровня тиреотропного гормона (ТТГ) [1].

Адекватное количество гормонов щитовидной железы у матери во время беременности необходимо для оптимального исхода беременности и нормального развития плода [2].

Нарушение функции щитовидной железы у матери (манифестный и субклинический гипо- и гипертиреоз) встречается в 2–4% случаев и ассоциировано с более высоким риском таких неблагоприятных исходов беременности, как выкидыш, преэклампсия, преждевременные роды, низкий интеллектуальный уровень ребенка [3].

Учитывая основные изменения в физиологии щитовидной железы, для оптимальной диагностики и лечения заболеваний щитовидной железы во время беременности необходима разработка референсных интервалов тиреоидных гормонов для конкретного населения, проживающего в различных условиях йодной обеспеченности [4][5].

## ЦЕЛЬ ИССЛЕДОВАНИЯ

Определить триместр-специфичные референсные интервалы для ТТГ у беременных женщин, проживающих в центральных регионах РФ, сопоставимых по степени тяжести дефицита йода.

## МАТЕРИАЛЫ И МЕТОДЫ

## Место и время проведения исследования

Место проведения.

Исследование проводилось на базе нескольких лечебных учреждений. Включение беременных женщин, сбор биоматериала, заполнение информированного согласия проводились на базе женских консультаций 3 медицинских организаций: Ивановский научно-исследовательский институт материнства и детства им. В.Н. Городкова, Смоленский государственный медицинский университет Министерства здравоохранения Российской Федерации, Городская клиническая больница им. В.В. Вересаева Департамента здравоохранения города Москвы. Лабораторный анализ проводился на базе Клинико-диагностической лаборатории ФГБУ «НМИЦ эндокринологии» Минздрава РФ.

Время исследования.

Сбор биоматериала проводился с августа 2018 г. по декабрь 2020 г. Затем биоматериал транспортировался в ФГБУ «НМИЦ эндокринологии» Минздрава РФ, где проводились лабораторные исследования и анализ полученных результатов.

## Изучаемые популяции

Беременные женщины, проживающие в 3 регионах РФ (Москва, Иваново, Смоленск).

Критерии включения: в исследование были включены здоровые беременные женщины на разных сроках беременности в возрасте от 18 до 45 лет. Всем беременным женщинам при первичной постановке на учет в женской консультации был рекомендован прием физиологических доз калия йодида 200 мкг в сутки.

Критерии исключения: прием препаратов, оказывающих влияние на функцию щитовидной железы, отказ пациентки от участия в исследовании (на любом этапе), многоплодная беременность, аномалия плода, тяжелая соматическая патология, тяжелые психические заболевания. Женщины, имеющие повышенный титр антител к тиреопероксидазе (АТ-ТПО) и/или антител к тиреоглобулину (АТ-ТГ) по результатам обследования исключались из исследования.

## Способ формирования выборки из изучаемой популяции

Сплошной способ формирования выборки.

## Дизайн исследования

Проведено наблюдательное одномоментное многоцентровое поперечное сплошное исследование.

## Описание медицинского вмешательства и методы

Всем женщинам, включенным в исследование, определялись следующие показатели сыворотки крови: ТТГ, АТ-ТПО, АТ-ТГ, исследовался уровень экскреции йода с мочой. Забор крови проводился рано утром, натощак. Кровь центрифугировали через 30–40 мин после сбора. Затем жидкую часть сыворотки перемещали в подготовленные пробирки, маркировали (номер пациента) и подвергали заморозке (менее -20°С) с последующей подготовкой для транспортировки в ФГБУ «НМИЦ эндокринологии» Минздрава РФ. Сбор мочи для определения йода проводился утром, в пластмассовые стаканы. Затем небольшое количество мочи (около 1 мл) переносили в пробирки типа эппендорф, маркировали (номер пациента) и подвергали заморозке с последующей подготовкой к транспортировке. Не менее чем за 1 сут пациентка не должна была использовать йод в качестве наружного средства.

Беременные женщины заполняли анкету, где отвечали на следующие вопросы: дата рождения, срок беременности, какие препараты йода получают.

Уровень ТТГ, АТ-ТПО определяли методом хемилюминесцентного иммуноанализа на автоматическом анализаторе ARCHITECT i2000 (Abbott). Референсные значения для ТТГ — 0,25–3,5 мМЕ/л, для АТ-ТПО — 0–5,6 МЕ/мл. Уровень АТ-ТГ определяли методом электрохемилюминесцентного анализа на автоматическом анализаторе Cobas 6000 (Roche Diagnostics). Референсные значения для АТ-ТГ — 0–115 МЕ/мл.

Церий-арсенитным методом оценивалась экскреция йода с мочой (мкг/л) с расчетом ее медианной концентрации.

## Статистический анализ

Статистический анализ данных проведен с помощью программы Microsoft Excel 2016 и программного пакета Statistica 13 (StatSoft, США). Были проведены подсчет и распределение по группам в зависимости от уровня йодурии. Методика расчета референсных интервалов по Хоффману применялась после устранения выбросов данных. После ликвидации выбросов для каждого маркера были определены совокупные частоты встречающихся значений с расчетом 2,5 и 97,5 процентилей. Результаты представлены в виде: медиана, 2,5 и 97,5 процентили. Статистически значимыми предполагались различия при р<0,05.

## Этическая экспертиза

Данная работа была одобрена локальным этическим комитетом ФГБУ «НМИЦ эндокринологии» Минздрава России 27.09.2017, протокол №17.

## РЕЗУЛЬТАТЫ

Сплошным методом исследования были обследованы 2008 беременных женщин. По результатам исследования были исключены женщины с носительством АТ-ТПО/АТ-ТГ (245 женщин).

Все женщины, включенные в данное исследование, были разделены по месту проживания (Москва, Иваново, Смоленск). После получения результатов мы сформировали 2 группы в зависимости от уровня экскреции йода с мочой (меньше и больше 150 мкг/л). Во всех группах проводилась оценка уровня ТТГ в зависимости от триместра беременности (табл. 1 и 2). В табл. 3 и 4 представлены результаты сравнения уровня ТТГ в зависимости от йодной обеспеченности.

**Table:** Таблица 1. Уровни ТТГ у беременных женщин с адекватной йодной обеспеченностью (йодурия более 150 мкг/л)Table 1. TSH levels in pregnant women with adequate iodine sufficiency (ioduria more than 150 µg/l)

	I триместр Me ТТГ [ 2,5; 97,5]	II триместр Me ТТГ [ 2,5; 97,5]	III триместр Me ТТГ [ 2,5; 97,5]	p-value1
Москва	n=46 Me 1,18 [ 0,13;3,36]	n=66 Me 1,74 [ 0,36;3,74]	n=119 Me 1,56 [ 0,47;3,66]	0,039
Смоленск	n=25 Me 0,98 [ 0,006;3,41]	n=64 Me 1,46 [ 0,20;3,20]	n=121 Me 1,38 [ 0,44;3,68]	0,020
Иваново	n=79 Me 0,98 [ 0,05;2,78]	n=50 Me 1,0 [ 0,47; 2,92]	n=104 Me 1,11 [ 0,33;2,85]	0,241

**Table:** Таблица 2. Уровни ТТГ у беременных женщин с легким йодным дефицитом (йодурия меньше 150 мкг/л)Table 2. TSH levels in pregnant women with mild iodine deficiency (iodine less than 150 mcg/l)

	I триместр Me ТТГ [ 2,5; 97,5]	II триместр Me ТТГ [ 2,5; 97,5]	III триместр Me ТТГ [ 2,5; 97,5]	p-value2
Москва	n=55 Me 1,40 [ 0,17;2,78]	n=123 Me 1,40 [ 0,38;3,78]	n=226 Me 1,46 [ 0,31;3,04]	0,724
Смоленск	n=49 Me 0,93 [ 0,24;3,00]	n=82 Me 1,34 [ 0,42;2,88]	n=151 Me 1,36 [ 0,19;3,04]	0,002
Иваново	n=132 Me 0,95 [ 0,11;2,93]	n=93 Me 1,21 [ 0,22;2,48]	n=179 Me 1,11 [ 0,07;2,59]	0,049

**Table:** Таблица 3. Сравнение уровней ТТГ в каждом триместре с различной йодной обеспеченностьюTable 3. Comparison of TSH levels in each trimester with different iodine availability

	I триместр, p-value	II триместр, p-value	III триместр, p-value
Москва	0,257	0,016	0,072
Смоленск	0,919	0,376	0,446
Иваново	0,766	0,129	0,299

**Table:** Таблица 4. Сравнение уровней ТТГ в группах с адекватной йодной обеспеченностью и легким йодным дефицитом (сравнение одномоментно всех триместров)Table 4. Comparison of TSH levels in groups with adequate iodine supply and mild iodine deficiency (simultaneous comparison of all trimesters)

Город	p-value
Москва	0,040
Смоленск	0,177
Иваново	0,721

Медианная концентрация йода в моче составила: в Москве — 106 мкг/л, в Иваново — 119 мкг/л, в Смоленске —134 мкг/л, это нормальная йодная обеспеченность, однако не соответствующая гестационной норме (выше 150 мкг/л) [6].

На рисунках 1–3 представлены значения ТТГ в зависимости от триместра беременности, региона проживания.

Нежелательные явления отмечены не были.

**Figure:**
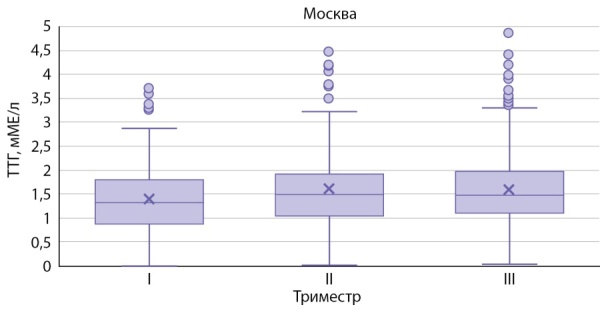
Рисунок 1. Уровень ТТГ у беременных женщин г. Москвы.Figure 1. TSH level in pregnant women in Moscow.

**Figure:**
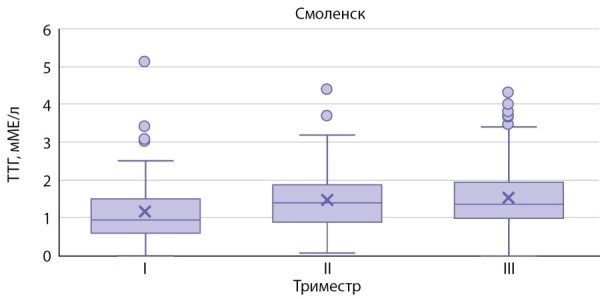
Рисунок 2. Уровень ТТГ у беременных женщин г. Смоленска.Figure 2. TSH level in pregnant women in Smolensk.

**Figure:**
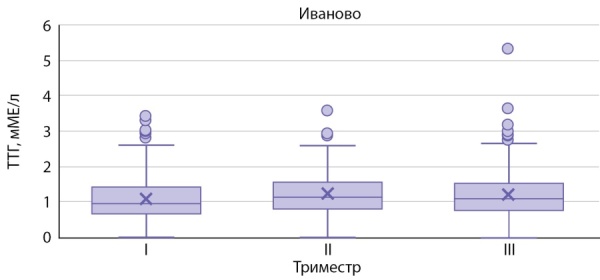
Рисунок 3. Уровень ТТГ у беременных женщин г. Иваново.Figure 3. TSH level in pregnant women in Ivanovo.

## ОБСУЖДЕНИЕ

## Репрезентативность выборок

Расчет размера выборки проведен в соответствии с техническими и иными возможностями представленных медицинских организаций по сбору и хранению биоматериала и составил от 320 до 450 женщин в год из каждого региона. Некоторые образцы были исключены из исследования в связи с ошибками при сборе и хранении. Также исследование проведено по принципу сплошного, а не выборочного исследования, что может являться обоснованием для отсутствия необходимости расчета минимального размера выборки.

## Сопоставление с другими публикациями

Вопрос о триместр-специфичных референсных значениях ТТГ у беременных женщин длительное время вызывает дискуссии в связи с тем, что исследования, проведенные на различных популяциях, не привели к однозначным выводам о верхнем и нижнем пределе ТТГ. В последних опубликованных клинических рекомендациях по диагностике и лечению заболеваний ЩЖ во время беременности экспертами было показано, что во время беременности происходит снижение как нижнего (на 0,1–0,2 мЕд/л), так и верхнего (на 0,5–1,0 мЕд/л) предела уровня ТТГ [4]. Экспертами были суммированы данные по эпидемиологическим исследованиям, проведенным на различных этнических группах с разным уровнем потребления йода. Так, в исследовании G. Lambert-Messerlian и соавт. (США) у беременных женщин, проживающих на территории легкого йодного дефицита, уровень ТТГ в I триместре был 0,12–3,37, во II триместре — 0,35–3,35 [7]. В исследовании S. La’ulu и соавт. (США), также проведенном на территории легкого йодного дефицита, уровень ТТГ в I триместре был значимо ниже — 0,02–2,69, во II — 0,15–3,11 [8][9]. Несмотря на то что оба исследования были проведены в США на территории легкого йодного дефицита, этнические составы групп были различными. В первом исследовании преобладала европейская этническая группа, тогда как во втором — испанская, а также афроамериканская. В исследованиях J. Bestwick и соавт., проведенных в Италии и Соединенном Королевстве, где на момент исследований был подтвержден йодный дефицит легкой и средней степени тяжести, верхний уровень ТТГ был 3,19 и 3,50 соответственно [10]. По данным исследования, опубликованного в 2005 г. на территории РФ, в Самарской области было проведено исследование на территории с дефицитом йода средней степени тяжести, в котором референсные интервалы ТТГ в I и II триместре были 0,09–4,67 и 0,20–4,68 соответственно [11]. В исследовании O. Bulur и соавт., проведенном на турецкой популяции, уровень ТТГ был 0,005–3,65 в I триместре, 0,011–3,63 — во II триместре, 0,2–3,46 — в III триместре [12]. Противоречивые данные были получены в исследованиях, проведенных на территориях без йодного дефицита. В исследовании С. Li и соавт. (китайская популяция) уровень ТТГ в I триместре был 0,10–4,34 [13]. В финской и нидерландской популяциях также было показано повышение верхнего предела уровня ТТГ (до 3,5 и 4,04 соответственно) [14][15].

В целом результаты нашего исследования соответствуют таковым, проведенным на территориях легкого йодного дефицита в европейских этнических группах. У исследуемых беременных женщин, получающих йодную профилактику и проживающих на территории легкого йодного дефицита, не выявлено повышение верхнего предела ТТГ выше 3,8 мМЕ/л. Уровень ТТГ по триместрам составил: в группе с оптимальным уровнем йодурии в I триместре — 0,006–3,36, во II триместре — 0,20–3,74, в III триместре — 0,33–3,68 мМЕ/л. В группе с легким йодным дефицитом: в I триместре — 0,11–3,00, во II триместре — 0,22–3,78, в III триместре — 0,07–3,04 мМЕ/л.

При сравнении уровня ТТГ в зависимости от уровня экскреции йода с мочой по регионам (суммарно по всем триместрам и по каждому триместру отдельно) статистически значимая разница уровня ТТГ получена только в Москве (p=0,04), в остальных регионах уровень ТТГ не отличался в группах сравнения.

## Клиническая значимость результатов

Впервые в РФ на большой выборке проведено исследование для определения триместр-специфичных референсных интервалов ТТГ у беременных женщин, проживающих на территории РФ с легким йодным дефицитом. Учитывая, что за последние несколько лет референсные интервалы уровня ТТГ претерпевали различные изменения, определение верхнего предела ТТГ необходимо для оценки показаний и целесообразности назначения терапии левотироксином натрия во время беременности.

## Ограничения исследования

Ограничением данного исследования является разное количество женщин в разделенных по триместрам группах, что обусловлено сплошным методом выборки.

## Направления дальнейших исследований

В дальнейшем необходимо провести исследование для определения референсных интервалов ТТГ на территориях со средним и тяжелым дефицитом йода.

## ЗАКЛЮЧЕНИЕ

Наше исследование показало, что референсные интервалы ТТГ у беременных женщин европейской расы, проживающих в центральных регионах РФ в условиях сохраняющегося легкого йодного дефицита, составили: в I триместре — 0,006–3,41 мМЕ/л, во II триместре — 0,20–3,78 мМЕ/л, в III триместре — 0,07–3,68 мМЕ/л.

Статистически значимых различий уровня ТТГ между регионами центральной части РФ получено не было, однако полученные данные нельзя экстраполировать на всю территорию РФ, в связи с чем необходимы дальнейшие исследования с расширением выборки с учетом этнических и экологических условий.

## ДОПОЛНИТЕЛЬНАЯ ИНФОРМАЦИЯ

Источник финансирования. Исследование выполнено за счет нескольких источников финансирования. Определение уровня ТТГ, АТ-ТПО, АТ-ТГ выполнено за счет средств гранта №22-15-00135 «Научное обоснование, разработка и внедрение новых технологий диагностики коморбидных йододефицитных и аутоиммунных заболеваний щитовидной железы, в том числе с использованием возможностей искусственного интеллекта». Определение уровня экскреции йода с мочой выполнено за счет НИР № АААА-А20-120011790180-4 «Эпидемиологические и молекулярно-клеточные характеристики опухолевых, аутоиммунных и йододефицитных тиреопатий как основа профилактики осложнений и персонализации лечения».

Конфликт интересов. Авторы декларируют отсутствие явных и потенциальных конфликтов интересов, связанных с публикацией настоящей статьи.

Участие авторов. Все авторы внесли значимый вклад в проведение поисково-аналитической работы и подготовку статьи, прочли и одобрили финальную версию до публикации.

Благодарности. Выражается благодарность за организацию и контроль проведения исследования: Ивановскому научно-исследовательскому институту материнства и детства им. В.Н. Городкова, Смоленскому государственному медицинскому университету Министерства здравоохранения Российской Федерации, Городской клинической больнице им. В.В. Вересаева Департамента здравоохранения города Москвы.
